# The current pathogens and treatment of hospital-acquired pneumonia/ventilator-associated pneumonia in medical intensive care units

**DOI:** 10.1186/2197-425X-3-S1-A707

**Published:** 2015-10-01

**Authors:** Y Chang, JY Moon, YJ Cho, SM Lee, K Jeon, SC Kim, YS Kim, YP Chong, YS Kim, SB Hong

**Affiliations:** Chungbuk National Univ. Hospital, Chungbuk National University College of Medicine, Division of Pulmonary and Critical Care Medicine, Department of Internal Medicine, Cheongju, Korea Republic of; Chungnam National Univ. Hospital, Chungnam National University College of Medicine, Division of Pulmonary and Critical Care Medicine, Department of Internal Medicine, Daejeon, Korea Republic of; Seoul National University Bundang Hospital, Seoul National University College of Medicine, Division of Pulmonary and Critical Care Medicine, Department of Internal Medicine, Seongnam, Korea Republic of; Seoul National University Hospital, Seoul National University College of Medicine, Division of Pulmonary and Critical Care Medicine, Department of Internal Medicine, Seoul, Korea Republic of; Samsung Medical Center, Sungkyunkwan University School of Medicine, Department of Pulmonary and Critical Care Medcine, Seoul, Korea Republic of; St.Mary's Hospital, The Catholic University of Korea, Division of Pulmonology, Department of Internal Medicine, Seoul, Korea Republic of; Asan Medical Center, University of Ulsan College of Medicine, Department of Infectious Diseases, Seoul, Korea Republic of; Severance Hospital, Yonsei University College of Medicine, Division of Pulmonology, Department of Internal Medicine, Seoul, Korea Republic of; Asan Medical Center, University of Ulsan College of Medicine, Department of Pulmonary and Critical Care Medicine, Seoul, Korea Republic of

## Introduction

Because of increasing prevalence of multi-drug resistant (MDR) pathogens, there are many difficulties in the treatment of hospital-acquired pneumonia (HAP)/ventilator-associated pneumonia (VAP). It is known that patients with MDR pathogens have high mortality and morbidity. We need to know the current status of pathogens and antibiotics therapy for a proper choice of initial antibiotics to improve outcome of the patients.

## Objectives

This study was aimed at investigating the current pathogens and treatment of HAP/VAP in Korean ICUs. Methods A prospective, multicenter and observational study was conducted in six tertiary referral hospitals in Korea between August 1, 2012 and July 31, 2013. This study included patients who were admitted to adult medical ICUs with diagnosed as pneumonia developing at least 48 hours after admission or initiation of mechanical ventilation. We analyzed the prevalence of their pathogens, and treatment and clinical courses of these patients.

## Results

During the study period of 12 months, total 134 patients with HAP/VAP were enrolled. Mean age was 65 with 103 (77%) males. Forty-seven (35%) patients had risk factors of MDR pathogens. The duration between admission and diagnosis of pneumonia was 12 days (IQR: 7-22 days). Most of patients (86%) underwent mechanical ventilation. Pathogens were identified in 79 (59%) patients. Among them, MDR bacteria were isolated in 55 (76%) patients. The most common MDR pathogen was carbapenem-resistant Acinetobacter baumannii (60%). However, extended-spectrum penicillin/β-lactamase inhibitor-based was the most common empirical antibiotics (39%) during initial 48 hours after diagnosis. Glycopeptides were used in 60 patients (47%). As a subsequent therapy between 48 hours and 2 weeks after diagnosis, carbapenem-based regimen was most common (36%). On the other hand, colistin-based therapy was done in 29 (22%) patients. Only 45 (34%) patients showed a clinical resolution within 28 days. They showed a high in-hospital mortality of 51% with 28-day and 60-day of 31% and 49%, respectively.

## Conclusions

This study demonstrates that carbapenem-resistant A. baumannii is the most common MDR pathogen in Korean medical ICUs. It shows that there is a discrepancy between the common MDR pathogen and the choice of empirical antibiotic therapy.
